# Reinforcement of Percutaneous Pedicle Screw Fixation with Hydroxyapatite Granules in Patients with Osteoporotic Spine: Biomechanical Performance and Clinical Outcomes

**DOI:** 10.3390/medicina58050579

**Published:** 2022-04-23

**Authors:** Haruo Kanno, Yoshito Onoda, Ko Hashimoto, Toshimi Aizawa, Hiroshi Ozawa

**Affiliations:** 1 Department of Orthopaedic Surgery, Tohoku Medical and Pharmaceutical University, Sendai 983-8536, Japan; hozawa@tohoku-mpu.ac.jp; 2 Department of Orthopaedic Surgery, Tohoku University School of Medicine, Sendai 980-8574, Japan; onodaworld@hotmail.com (Y.O.); hasshie@gmail.com (K.H.); toshi-7@med.tohoku.ac.jp (T.A.)

**Keywords:** percutaneous pedicle screw, hydroxyapatite granules, augmentation, osteoporosis, spine, screw loosening, spine surgery, minimally invasive spinal treatment, minimally invasive spine stabilization

## Abstract

In percutaneous pedicle screw (PPS) fixation of the osteoporotic spine, rigid screw fixation obtaining strong stabilization is important for achieving successful treatment outcomes. However, in patients with severe osteoporosis, it is difficult to obtain PPS fixation with sufficient stability. PPS fixation has potential disadvantages with respect to maintaining secure stabilization in comparison to conventional pedicle screw fixation. In PPS fixation, bone grafting to achieve posterior spine fusion is generally not applicable and transverse connectors between the rods cannot be used to reinforce the fixation. Various augmentation methods, including additional hooks, sublaminar bands, and hydroxyapatite (HA) sticks, are available for conventional pedicle screw fixation. On the other hand, there has been no established augmentation method for PPS fixation. Recently, we developed a novel augmentation technique for PPS fixation using HA granules. This technique allows the percutaneous insertion of HA granules into the screw hole along the guidewire prior to insertion of the PPS. We have used this augmentation technique for PPS fixation in various spine surgeries in patients with osteoporosis. In our previous studies, biomechanical analyses demonstrated that PPS fixation was significantly enhanced by augmentation with HA granules in the osteoporotic lumbar spine. Furthermore, augmentation with HA granules was considered to decrease the incidence of screw loosening and implant failure following PPS fixation in patients with osteoporotic spine. In this article, we describe the surgical procedures of the augmentation method using HA granules and summarize our data from the biomechanical analysis of augmentation for PPS fixation. We also review the surgical outcomes of PPS fixation with augmentation using HA granules.

## 1. Introduction

In the last decade, percutaneous pedicle screw (PPS) fixation has been widely used for minimally invasive spinal surgery. PPS fixation has been performed for various surgeries to treat spinal trauma, tumors, infection, deformity, and degenerative diseases in the thoraco-lumbar spine [1]. PPS fixation can reduce the damage of the surrounding tissues, intraoperative blood loss, postoperative pain, and recovery time in comparison to conventional pedicle screw fixation [2,3,4].

Osteoporosis is becoming more common as the population ages [5,6]. In pedicle screw fixation for osteoporotic spine, rigid screw fixation and strong stabilization are essential for achieving successful treatment outcomes. However, in osteoporotic patients, it is difficult to obtain screw fixation with sufficient stability due to bone fragility. Therefore, there are certain risk factors for screw loosening and implant failure after surgery with instrumentation for osteoporotic spine [7,8]. Various augmentation methods have been used for conventional pedicle screw fixation in spine surgery for patients with osteoporosis, including additional hooks [9,10,11], sublaminar bands [12,13], cement augmentation [14,15], and hydroxyapatite (HA) sticks [16,17].

PPS fixation has potential disadvantages with respect to maintaining strong stabilization in comparison to conventional pedicle screw fixation. In PPS fixation, bone grafting to achieve posterior spine fusion is generally not applicable [18,19]. Because of the percutaneous technique involving a small incision, transverse connectors between the rods cannot be used to reinforce the PPS fixation [20,21]. Conventional augmentation methods, including additional hooks and sublaminar bands, are not applicable in PPS fixation. However, there has been no standardized augmentation method for PPS fixation.

Recently, we developed a novel augmentation technique for PPS fixation using hydroxyapatite (HA) granules [22]. This technique allows for the percutaneous insertion of HA granules into the screw hole along the guidewire prior to insertion of the PPS [22,23]. We have used this augmentation technique for PPS fixation in various spine surgeries [23,24]. The biomechanical analyses of our previous studies demonstrated that PPS fixation was significantly enhanced by augmentation with HA granules in the osteoporotic lumbar spine [22,23]. Furthermore, augmentation with HA granules was considered to reduce the incidence of screw loosening and implant failure following PPS fixation in patients with osteoporotic spine [22,24,25].

In this article, we describe the surgical procedures of the augmentation method using HA granules and summarize the data obtained from the biomechanical analysis of augmentation for PPS fixation. We also review the surgical outcomes of PPS fixation with augmentation using HA granules.

## 2. Surgical Procedure of Augmentation of PPS Fixation Using HA Granules

**Figure 1:**
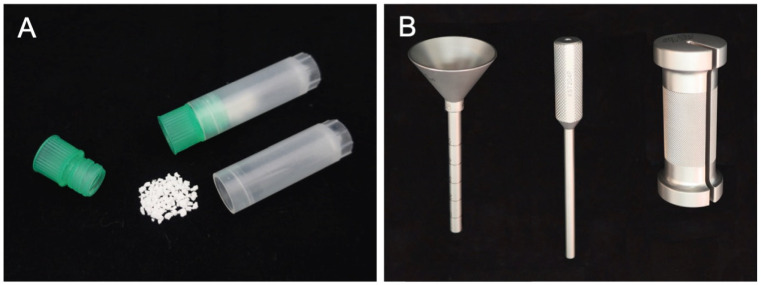
HA granules and the dedicated inserter for augmentation of PPS fixation. HA granules (porosity, 50%; particle size, 1–2 mm) are used for augmentation (**A**). The inserter consists of a funnel-shaped external cylinder, internal cylinder, and slide hammer (**B**).

In our augmentation technique for PPS fixation, HA granules can be inserted percutaneously into the screw hole along the guidewire using a dedicated inserter (Figure 1). This method has the advantage that PPS fixation can be enhanced percutaneously without compromising the minimally invasive procedure. We use commercially available HA granules (porosity, 50%; particle size, 1.0–2.0 mm; Apaceram, HOYA Technosurgical Corp., Tokyo, Japan) for the augmentation (Figure 1A) [22,23]. Based on the results of biomechanical analyses in our previous studies [22,23], at least 0.25 g of HA granules are used for the augmentation for each PPS. We created a dedicated device to insert the HA granules percutaneously into the screw hole in order to achieve the augmentation (Figure 1B) [14].

**Figure 2:**
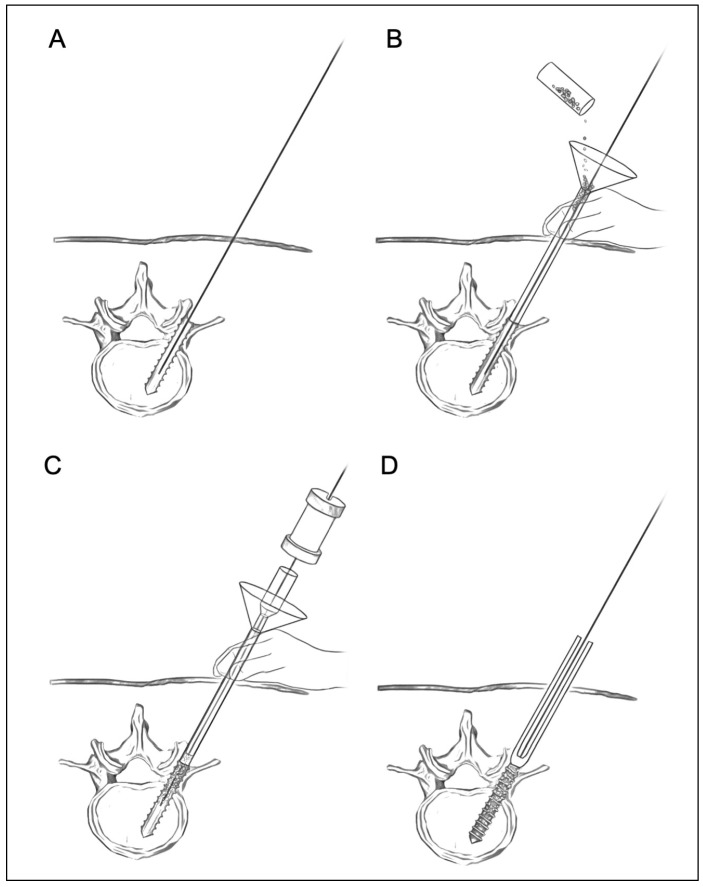
Surgical procedures for augmentation of PPS fixation using HA granules. A guidewire is inserted into the vertebra, and then tapping is performed (**A**). The funnel-shaped external cylinder is placed at the screw hole along the guidewire. HA granules are put into the external cylinder (**B**). Then, the HA granules are pushed into the screw hole using the internal cylinder and slide hammer (**C**). Finally, screw insertion is performed (**D**).

**Figure 3:**
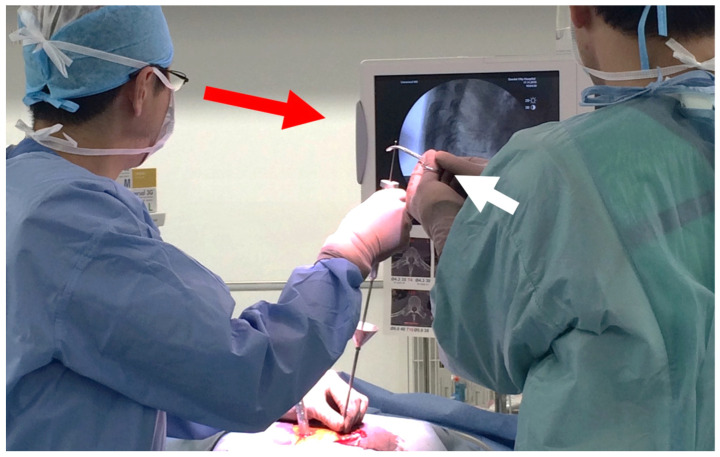
HA granules are pushed into the screw hole using the internal cylinder and slide hammer. The position of the tip of the guidewire must be carefully checked on a lateral fluoroscopic image in order to prevent the guidewire from penetrating the anterior wall of the vertebral body (red arrow). In addition, an assistant should grasp securely the proximal part of the guidewire with a Kocher forceps to prevent the guidewire from moving forward (white arrow).

The surgical procedures for the augmentation of PPS fixation are as follows (Figure 2). First, according to the standard method of PPS insertion, the guidewire is inserted into the pedicle and posterior part of the vertebral body using a Jamshidi needle under fluoroscopic guidance. Then, the screw hole is prepared using a cannulated tap along the guidewire (Figure 2A). Secondly, the funnel-shaped external cylinder of the insertion device is placed at the screw hole along the guidewire (Figure 2B). Then, HA granules are inserted into the external cylinder. The tip of the inserter was set at the entry point of the screw hole so that the HA granules were mainly placed within the pedicle but not the vertebral body. Using the internal cylinder and slide hammer, the HA granules are pushed into the screw hole (Figure 2C). The position of the tip of the guidewire must be carefully checked on a lateral fluoroscopic image in order to prevent the guidewire from penetrating the anterior wall of the vertebral body (Figure 3). In addition, an assistant should securely grasp the proximal part of the guidewire with a Kocher forceps to prevent the guidewire from moving forward (Figure 3). After removing the insertion device, the PPS can be inserted into the screw hole along the guidewire (Figure 2D).

## 3. Biomechanical Analysis of Augmentation of PPS Fixation Using HA Granules

Previous studies indicated that the placement of substances into the tapped screw hole increases the bone–metal interface friction force and enhances the mechanical strength of screw fixation [16,26,27,28,29,30,31]. We previously performed a biomechanical analysis to evaluate the strength and stiffness of PPS fixation augmented with HA granules using a synthetic bone model [22]. The results of that study demonstrated the biomechanical advantages of augmentation with HA granules for PPS fixation in the osteoporotic bone model. The study showed that the maximal insertion torque and the maximal pullout strength were significantly increased in screws with augmentation in comparison to without augmentation. Furthermore, the mechanical strength against cyclic loading was significantly greater in screws with augmentation in comparison to those without augmentation.

In another study, we performed a cadaveric biomechanical analysis of PPS fixation augmented with HA granules [23]. The biomechanical performance in augmenting PPS fixation was evaluated using osteoporotic lumbar vertebrae obtained from cadavers. Our results demonstrated that the augmentation using HA granules significantly increased the maximal pullout strength and maximal insertion torque of the screws placed in the osteoporotic lumbar spine. Moreover, a cyclic loading test revealed that the augmented screws achieved significantly higher mechanical strength. 

These findings suggest that PPS fixation can be enhanced by augmentation with HA granules in the osteoporotic lumbar spine. PPS fixation augmented with HA granules may be helpful for decreasing the incidence of screw loosening and implant failure in patients with osteoporotic spine.

## 4. Postoperative Stability of PPS Fixation in Osteoporotic Patients

To determine whether augmentation with HA granules can improve the postoperative stability of PPS fixation in osteoporotic patients, we previously evaluated the incidence of screw loosening and implant failure after surgery [25]. In this study, we analyzed 32 patients with osteoporotic spine (male, *n* = 18; female, *n* = 14; age, 74 ± 11 years) who underwent PPS fixation with augmentation using HA granules at multiple levels (5.9 ± 1.9 levels). Postoperative screw loosening was assessed by radiographic images and the presence of a clear zone around the screw on X-ray and computed tomography (CT) [32,33]. The presence or absence of reoperation due to postoperative implant failure was also investigated. In our results, screw loosening was observed in 21 of 360 screws (5.8%) and 8 of 32 patients at the final follow-up examination (15 ± 9.8 months). Importantly, there were no cases of reoperation due to implant failure. No patients had cardiovascular or neurological complications associated with augmentation with HA granules.

Ohtori et al. showed that 26 of 102 (25.5%) conventional pedicle screws implanted in patients with osteoporotic spine became loose at the 12-month follow-up examination [33]. In addition, Ohba et al. reported that screw loosening was found in 44 of 290 screws (15.2%) at 1 year after PPS fixation [32]. In the results of our study, the incidence of screw loosening (5.8%) was lower than that in previous reports. Thus, augmentation with HA granules may improve the stability of PPS fixation and consequently decrease the incidence of postoperative screw loosening and implant failure.

## 5. Illustrative Case

**Figure 4:**
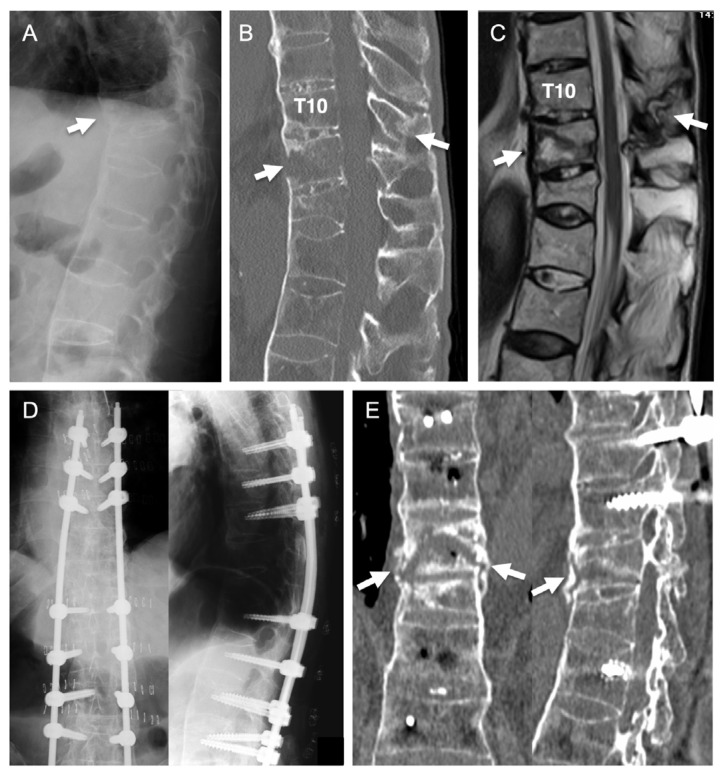
Illustrative case. Preoperative images showed spinal ankylosis and spinal fracture at the T10–11 levels (arrows in (**A**–**C**)). PPS fixation with augmentation using HA granules was performed (**D**). Bone union at the fracture site was achieved at 12 months after surgery (arrows in (**E**)).

**Figure 5:**
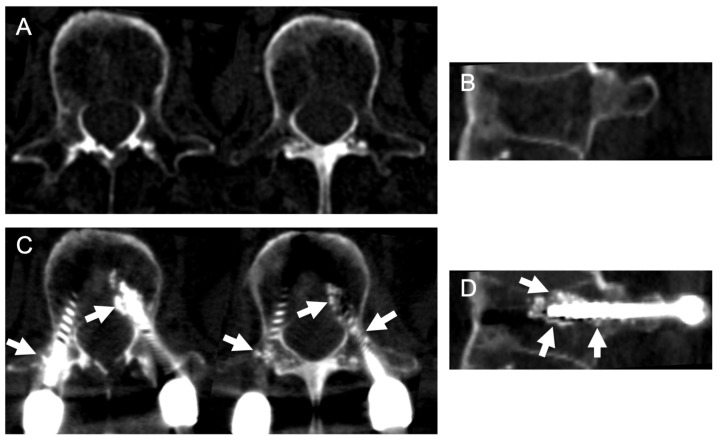
Computed tomography before (**A**,**B**) and after (**C**,**D**) surgery. Postoperative axial (**C**) and sagittal (**D**) images revealed that the HA granules surrounded the screws within the pedicle and vertebral body (Arrows).

The patient was an 81-year-old man with ankylosing spondylitis and severe osteoporosis. He fell from a stepladder and sustained a thoracic spinal fracture. He had been hospitalized for one month at another hospital. However, his severe back pain did not improve and he was confined to bed. He was then referred to our department for further treatment. An imaging study at our hospital revealed spinal ankylosis and spinal fracture at the T10–11 level (Figure 4A–C). The patient underwent surgery to perform PPS fixation with augmentation using HA granules (Figure 4D). After the operation, his symptoms disappeared and he became able to walk without any support. Postoperative CT revealed HA granules surrounding the screws within the pedicle and vertebral body (Figure 5). Bone union at the fracture site was achieved at 12 months after surgery (Figure 4E). There was no implant failure or screw loosening in the postoperative course.

## 6. Discussion

Spinal surgery with instrumentation in patients with osteoporotic spine is challenging because of their bone fragility [34]. When pedicle screw fixation is performed in patients with osteoporosis, the incidence of loosening or screw back-out is higher [7,8]. Therefore, rigid screw fixation and secure stabilization of the spine are crucial for preventing postoperative implant failure. However, conventional augmentation methods, such as supplemental hooks and sublaminar bands, are normally not applicable to PPS fixation due to the percutaneous surgical procedure [18,19]. In PPS fixation, it is very difficult to insert biomaterials, such as HA sticks and bone cement, into the tapped pedicle because surgeons cannot see the screw hole directly and because a guidewire is placed within the pedicle percutaneously. Importantly, the use of an insertor for HA granules enables PPS augmentation to be achieved percutaneously in a minimally invasive procedure without additional skin incision [22,23]. The data of the biomechanical analysis demonstrated that PPS fixation with augmentation using HA granules produced significantly stronger screw pullout strength and insertion torque [22,23]. In addition, the reinforcement of the screws with HA granules significantly enhanced the resistance to cyclic loads. Furthermore, augmentation with HA granules decreased the incidence of screw loosening in osteoporotic patients after PPS fixation [24,25]. Augmentation using HA granules can be a practical method for minimally invasive spinal surgery with PPS fixation in patients with osteoporosis.

The reinforcement of pedicle screws using polymethylmethacrylate (PMMA) can increase the fixation strength of spinal instrumentation in the osteoporotic spine [15]. A previous study indicated that pedicle screws augmented by PMMA cement showed significantly stronger pullout strength in comparison to non-augmented screws [35]. However, the augmentation method using PMMA cement has several unfavorable issues. PMMA is unable to induce bone remodeling, osteoinduction, osteoconduction, or osteointegration and its presence can inhibit the vascular supply. PMMA cement has exothermic properties that may induce bone necrosis and degeneration of the adjacent discs [36,37]. Furthermore, augmentation with PMMA cement causes a risk of vertebral fracture that can induce nerve root or dural injury during screw removal [38]. In contrast, HA has high bioaffinity and biocompatibility, which induces osteoconduction and osteointegration [39]. HA can be slowly replaced by the host bone [39]. HA induces no exothermic reaction or toxic effects that could damage the surrounding bone or soft tissue following implantation. 

Cement-based augmentation is associated with a risk of cement extravasation, which may cause cardiovascular complications [36]. The insertion of PMMA cement into the vertebral body has been widely recognized as a possible cause of pulmonary cement embolism and fat embolism syndrome [40,41,42,43]. Many previous reports have indicated that percutaneous vertebroplasty and balloon kyphoplasty using PMMA is associated with a certain risk of embolic events [44,45]. Indeed, it has been reported that augmentation of pedicle screws with PMMA cement caused severe fat embolism syndrome [46]. Over the past few decades, the augmentation of pedicle screws using HA granules has been widely used in spinal surgeries in various countries [16,17,22,47]. In addition, vertebroplasty with transpedicular HA block grafting has long been performed for the treatment of thoracolumbar vertebral fracture [48,49,50]. However, to our knowledge, there have been no previous reports of symptomatic or asymptomatic pulmonary embolism associated with the augmentation of pedicle screws using HA granules. Only one study reported a case of asymptomatic pulmonary embolism after vertebroplasty with HA ceramic blocks [51]. It has been suggested that the insertion of solid material into the vertebral body may be safer than injecting liquid material with respect to the risk of extravertebral leakage causing embolism [40,51]. Therefore, the insertion of HA granules into the vertebral body may be associated with a lower risk of embolic events in comparison to the injection of PMMA cement [52]. Although the potential risk of an embolic event during spinal surgery should not be ignored, HA granules are considered safer than PMMA cement for the augmentation of PPS fixation.

## 7. Conclusions

The rigidity and stability of PPS fixation can be enhanced by augmentation with HA granules in the osteoporotic spine. Augmentation with HA granules may help to reduce the incidence of screw loosening and implant failure following PPS fixation. Augmentation using HA granules can be a practical method for minimally invasive spinal surgery with PPS fixation in patients with osteoporosis.
